# Cranial Suture Joints in Sheep *Ovis aries* (*Najdi breed*): Anatomical and Radiographic Findings

**DOI:** 10.3390/vetsci13050416

**Published:** 2026-04-24

**Authors:** Fahad Abdullah Alshanbari, Gamal Mounir Allouch, Madeh Sadan, Moustafa Salouci

**Affiliations:** 1 Department of Medical Biosciences, College of Veterinary Medicine, Qassim University, Buraydah 51452, Saudi Arabia; g.alloush@qu.edu.sa; 2 Department of Clinical Sciences, College of Veterinary Medicine, Qassim University, Buraydah 51452, Saudi Arabia; m.sadan@qu.edu.sa; 3 Department of Anatomy, Collage of Veterinary Medicine, King Faisal University, Al-Ahsa 31982, Saudi Arabia; msalouci@kfu.edu.sa

**Keywords:** animals, cranium, morphology, skull, sutures, sheep

## Abstract

This study aimed to describe the anatomical and morphological features of cranial suture joints in sixteen sheep skulls and to compare these findings with those reported in other domesticated species. The study of cranial sutures extends beyond morphology to elucidate mechanisms of skull growth, as suture patterns function as regulatory units in cranial development, highlighting the important roles in comparative and evolutionary studies, age estimation, and fracture identification, and serving as reference points in veterinary surgery and radiography. A detailed anatomical examination of sheep skulls was performed to identify and classify cranial sutures based on their morphology and anatomical location. Sutures were categorized according to their structural patterns and regional distribution within the cranium. Twenty-eight cranial sutures were identified and classified into four morphological types: serrated, plane, squamosal and foliate. These sutures were distributed across the dorsal, ventral, lateral–vertical, and internal cranial regions, reflecting the complex organization of cranial bones, indicating intraspecies diversity influenced by species-specific cranial architecture and developmental factors. The absence of certain sutures, such as the sagittal suture in sheep, was found. This study provides a detailed anatomical reference for cranial sutures in sheep. In sheep, suture morphology is closely related to the skull’s adaptation to mechanical stresses during growth, including forces generated by fighting and self-defense behaviors and high chewing mechanics. The findings offer valuable baseline data for veterinary clinicians and anatomists in distinguishing normal anatomical patterns from congenital cranial abnormalities.

## Simple Summary

This study examined sixteen adult Najdi sheep skulls (1–2 years old) prepared through cleaning, chemical treatment, and drying, followed by anatomical and radiographic evaluation of cranial sutures. The results showed that sheep cranial sutures are classified into four types—planar, serrate, squamosal, and foliate—each differing in structure and location. Planar sutures are smooth and mainly provide stability during mastication, while serrate sutures are interlocking and adapted for shock absorption during behaviors such as head-butting. Squamosal sutures have overlapping edges that help distribute mechanical forces and protect deeper structures, whereas foliate sutures exhibit complex, leaf-like patterns that enhance skull strength and protection. Overall, the morphology of these sutures reflects functional, biomechanical, and behavioral adaptations in sheep.

## 1. Introduction

The skull is a complex bony structure forming the head and is typically described as an irregular pyramidal shape with five surfaces. In many animal species, it is composed of 36 distinct bones, including both paired and unpaired elements [1]. These bones are interconnected by fixed, immobile joints known as sutures, which occur primarily between the flat bones of the skull [2,3,4]. In sheep, several cranial sutures have been documented in previous anatomical investigations; however, comprehensive identification of these sutures remains incomplete. Moreover, existing descriptions are frequently brief and provide limited detail regarding sutural morphology and structural characteristics [5]. Cranial sutures are formed by the articulating margins of adjacent skull bones and are distributed throughout the cranium in mammals, where they exhibit marked morphological variability and differing degrees of interdigitation, both among different species and within a single species [6,7].

Beyond their structural role in connecting adjacent bones, cranial sutures represent primary sites of osteogenesis during skull development [3,8]. Most sutures are named according to the bones they unite, while some possess distinct designations, such as the coronal suture, which connects the frontal and parietal bones [9]. Furthermore, previous studies have indicated that suture morphology reflects both anatomical and functional adaptations of the skull [10,11]. Despite their importance, information on fibrous joints, particularly cranial sutures in the sheep skull, remains limited. Therefore, the aim of the present study was to describe the fibrous suture junctions of the sheep skull, document their morphological variations, and compare these findings with those reported in other domesticated animal species. The results are expected to enhance our understanding of cranial suture patterns, support osteological research, and provide a reference framework for future anatomical and comparative studies.

## 2. Materials and Methods

This study was conducted on sixteen adult healthy Najdi sheep skulls of both sexes, aged 1–2 years, obtained from the Veterinary Anatomy Laboratory, Department of Medical Biosciences, College of Veterinary Medicine, Qassim University. The skulls were prepared by removing the skin, musculature, and surrounding soft tissues, after which they were boiled in water containing 30% sodium hydroxide (NaOH, SABIC, Riyadh, Saudi Arabia) for 4–8 h. Following boiling, the specimens were air-dried at room temperature for 48 h. For further cleansing, the skulls were immersed in 33% hydrogen peroxide for 48 h and subsequently dried for an additional three days, in accordance with previously described protocols [12,13,14,15]. Cranial sutures were then examined through detailed anatomical assessment to document their morphology and configuration. High-resolution photographs were obtained from mediolateral, anteromedial, ventrodorsal, and dorsoventral views.

**Radiographic examination:** Two standard projections, dorsoventral and lateral, were obtained for each skull. Imaging was performed using a Minx Ray HF 100/30 unit (Toshiba, Tokyo, Japan), and the resulting images were interpreted subjectively.

## 3. Results

Cranial sutures form along the adjoining margins of skull bones, and the morphology of these articulations varies among individual bones as well as across animal species. In the sheep skull, cranial sutures are classified into four main types: planar, serrate, squamosal, and foliate. The present study specifically addresses the classification and anatomical characterization of cranial planar sutures.

The selection of comparative taxa for describing cranial sutures in this study was based on scientific criteria aimed at clarifying functional, biomechanical, and evolutionary differences within the skull. Owing to the strong association between suture morphology and function, three criteria were applied. The morphological criterion focuses on suture geometry, including serrated, plane, squamous, and foliate types, which reflect adaptations for bone interlocking and mechanical resistance. The functional and biomechanical criterion considers compressive forces arising from behaviors such as self-defense; in species like sheep, increased suture complexity represents an adaptation that enhances stress distribution. Finally, the evolutionary criterion addresses interspecific variation in suture morphology among ruminants, carnivores, and equids, reflecting differences in diet and behavior.

### 3.1. First Type: Cranial Planar Sutures (Table 1)

#### 3.1.1. Internasal Planar Suture (Sutura Plana Internasalis) (Figure 1(1))

This unpaired, linear suture arises from the apex of the nasal bones and extends caudally toward the frontal suture. It lies along the rostral midline of the facial skeleton and unites the paired nasal bones.

**Table 1:** List of Cranial Planar Sutures with Corresponding Bone Articulations.

First Type: Cranial Planar Sutures		
No.	Planar Suture Name	Bones Involved
1	Internasal planar suture	Right and left nasal bones
2	Frontal planar suture	Right and left frontal bones
3	Incisivo-maxillary planar suture	Incisive bone and maxillary bone
4	Naso-maxillary planar suture	Nasal bone and maxillary bone
5	Lacrimo-zygomatic planar suture	Lacrimal bone and zygomatic bone
6	Nasolacrimal planar suture	Nasal bone and lacrimal bone
7	Fronto-lacrimal planar suture	Frontal bone and lacrimal bone
8	Tympano-mastoid planar suture	Tympany and mastoid bone
9	Zygomatico-maxillary planar suture	Zygomatic bone and maxillary bone
10	Median palatal planar suture	Right and left palatine bones
11	Pterygo-sphenoid planar suture	Pterygoid bone and sphenoid bone
12	Spheno-occipital planar suture	Sphenoid bone and occipital bone

**Figure 1:**
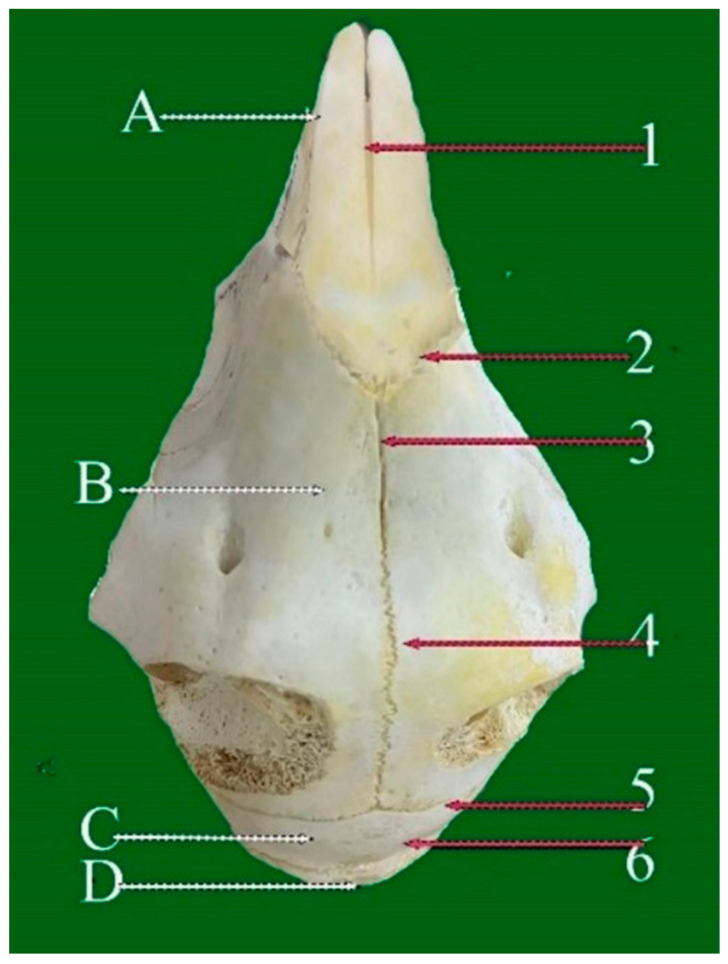
Dorsal view of sheep skull showing: 1—Internasal planar suture. 2—Naso-frontal foliate suture. 3—Frontal planar suture. 4—Frontal serrata suture. 5—Fronto-parietal suture (Squamosal carnii). 6—Occipito-parietal foliate suture. A: Nasal bone. B: Frontal bone. C: Partial bone. D: Occipital bone. (Please note that white refers to the bone and red arrow refers to the suture).

#### 3.1.2. Frontal Planar Suture (Sutura Plana Frontalis) (Figure 2(2))

Longer and straighter than the internasal suture, this horizontal suture connects the left and right frontal bones and runs in a caudorostral direction. It comprises two distinct regions: the rostral two-thirds display a planar configuration, whereas the caudal one-third exhibits a serrated pattern. Originating near the coronal suture at the horn bases, it gradually transitions from serrated to planar as it extends rostrally, terminating at its junction with the internasal suture along the midline.

**Figure 2:**
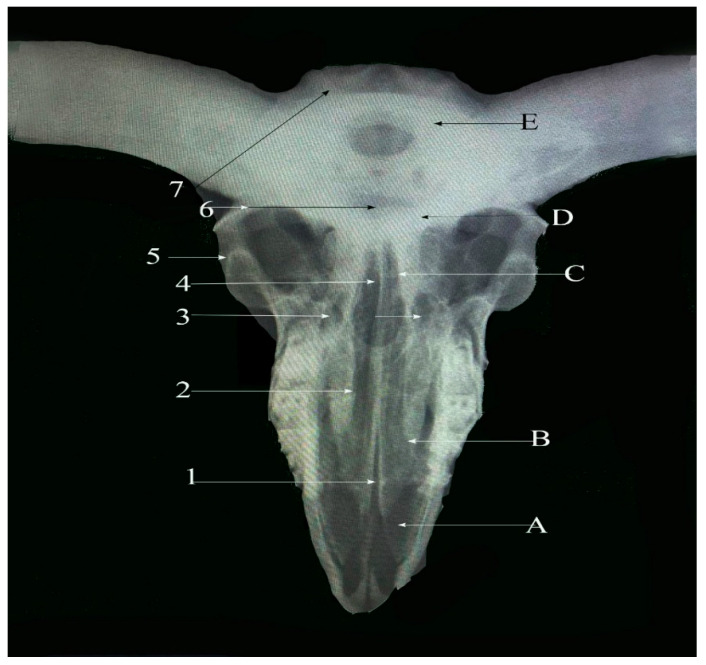
Dorsoventral view of sheep skull showing: 1—Internasal planar suture. 2—Frontal planar suture. 3—Naso-frontal foliate suture. 4—Frontal serrata suture. 5—Zygomatico-temporal planar suture. 6—Fronto-parietal suture (Squamosal carnii). 7—Occipito-parietal foliate suture. A: Nasal bone. B: Maxillary bone. C: Frontal bone. D: Partial bone. E: Occipital bone.

#### 3.1.3. Incisivo-Maxillary Planar Suture (Sutura Plana Incisive-Maxillaris) (Figure 3(1,4))

This curved and irregular suture joins the palatal processes of the incisive and maxillary bones on the palatal surface. Dorsolaterally, it merges with the naso-maxillary suture.

**Figure 3:**
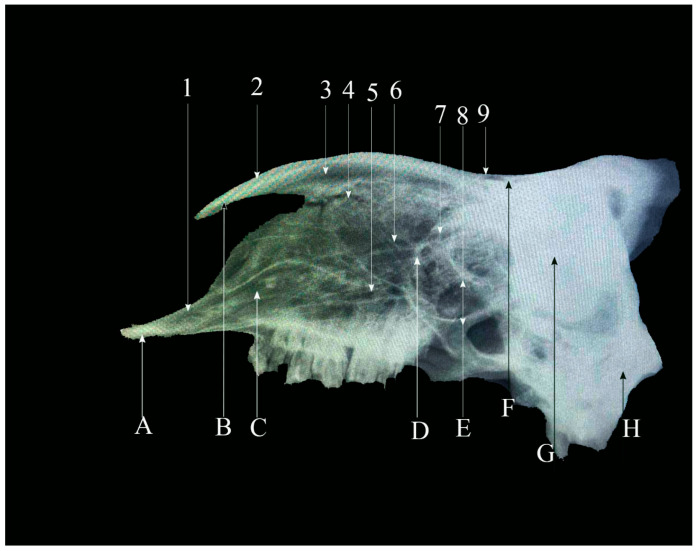
Lateral view of cranial sheep skull showing: 1—Incisive-maxillary planar suture. 2—Internasal planar suture. 3—Naso-maxillary planar suture. 4—Incisive-maxillary serrata suture. 5—Zygomatico-maxillary serrate suture. 6—Lacrimo-maxillary serrate suture. 7—Lacrimo-frontal serrata suture. 8—Zygomatico-temporal foliate suture. 9—Naso-frontal foliate suture. A: Incisive bone. B: Nasal bone. C: Maxillary bone. D: Lacrimal bone. E: Orbit. F: Frontal bone. G: Parietal. H: Occipital bone.

#### 3.1.4. Naso-Maxillary Planar Suture (Sutura Plana Naso-Maxillaris) (Figure 3(3) and Figure 4(2))

This paired planar suture delineates the junction between the lateral margin of the nasal bone and the frontal process of the maxilla. Dorsally, it articulates horizontally with the nasal bone.

**Figure 4:**
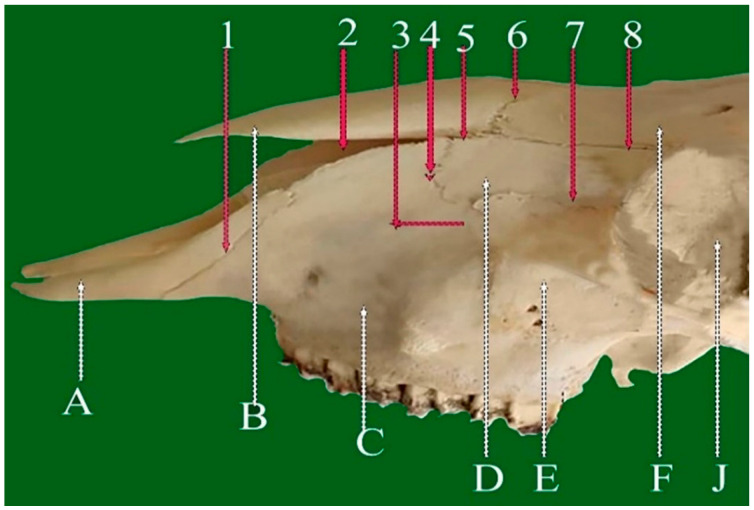
Lateral view of cranial sheep skull showing: 1—Incisive-maxillary (premaxilla) planar suture. 2—Naso-maxillary planar suture. 3—Zygomatico-maxillary (vertical part) serrate suture. 4—Lacrimo-zygomatic planar suture. 5—Nasolacrimal planar suture. 6—Naso-frontal foliate suture. 7—Lacrimo-maxillary serrate suture. 8—Fronto-lacrimal planar suture. A: Incisive bone. B: Nasal bone. C: Maxillary bone. D: Lacrimal bone. E: Zygomatic bone. F: Frontal bone. J: Orbit.

#### 3.1.5. Lacrimo-Zygomatic Planar Suture (Sutura Plana Lacrimo-Zygomaticus) (Figure 4(4))

This suture connects the lacrimal and zygomatic bones and runs parallel to the fronto-lacrimal suture. Extending along the nasolacrimal fissure, it terminates dorso-rostrally at its junction with the zygomatic-maxillary suture. Within the orbit, it follows the ventromedial border of the zygomatic bone and serves as an important orbital landmark.

#### 3.1.6. Nasolacrimal Planar Suture (Sutura Plana Nasolacrimalis) (Figure 4(5))

Situated between the nasal and lacrimal bones, this short planar suture represents a continuation of the fronto-lacrimal suture. It extends toward the naso-maxillary notch and may appear fissure-like in some specimens.

#### 3.1.7. Fronto-Lacrimal Planar Suture (Sutura Plana Fronto-Lacrimalis) (Figure 4(8))

It represents a key component of the orbital and facial skeleton in sheep. Located at the junction of the frontal and lacrimal bones, this suture is predominantly smooth with mild serrations. It courses dorsally along the orbital margin, extends rostrally toward the nasolacrimal fissure, and contributes to the stabilization of adjacent facial structures.

#### 3.1.8. Tympano-Mastoid Planar Suture (Sutura Plana Tympano-Mastoidea) (Figure 5(3))

Located between the caudal margin of the tympanic bone and the jugular process of the occipital bone, this suture represents a medial continuation of the tympano-squamosal suture.

**Figure 5:**
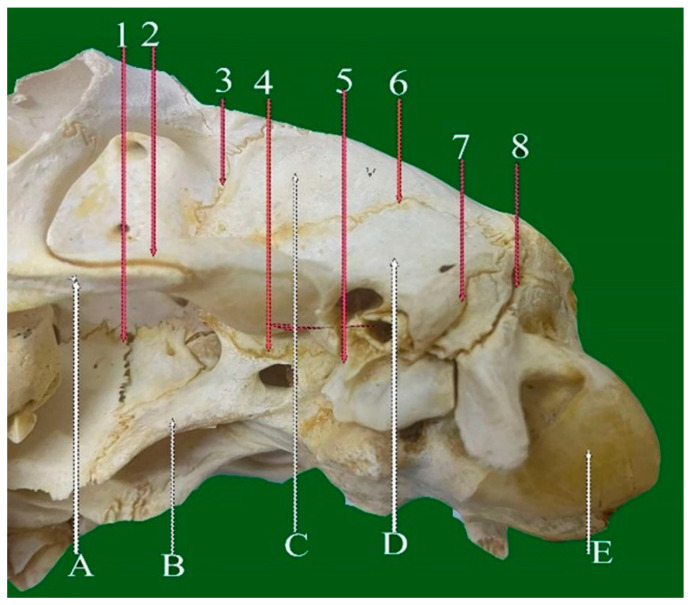
Ventrolateral view of sheep skull showing: 1—Pterygo-palatine serrate suture. 2—Zygomatico-temporal foliate suture. 3—Fronto-parietal serrate suture (coronal serrate suture). 4—Petro-squamous suture. 5—Tympano-mastoid planar suture. 6—Parieto-temporal (squamosal cranii) suture. 7—Tympano-mastoid suture. 8—Occipito-temporal foliate suture. A: Zygomatic bone. B: Pterygoid bone. C: Temporal bone. D: Parietal bone. E: Occipital bone.

#### 3.1.9. Zygomatico-Maxillary Planar Suture (Sutura Plana Zygomatico-Maxillaris) (Horizontal Part) (Figure 6(4) and Figure 7(6b))

The zygomatico-maxillary planar suture consists of the horizontal and the short vertical suture. The horizontal planar suture forms the articulation between the maxilla and zygomatic bone beneath the orbit, and the horizontal component runs in a caudorostral direction, typically along the infraorbital margin, forming part of the ventral boundary of the orbit and playing an important role in maintaining facial width and orbital stability.

Whereas the short vertical suture is serrated, extending dorsoventrally along the lateral aspect of the face, this vertical serrate suture connects part of the caudal border of the maxilla with the rostral edge of the zygomatic bone, enhancing the mechanical strength and stability of the orbital region.

**Figure 6:**
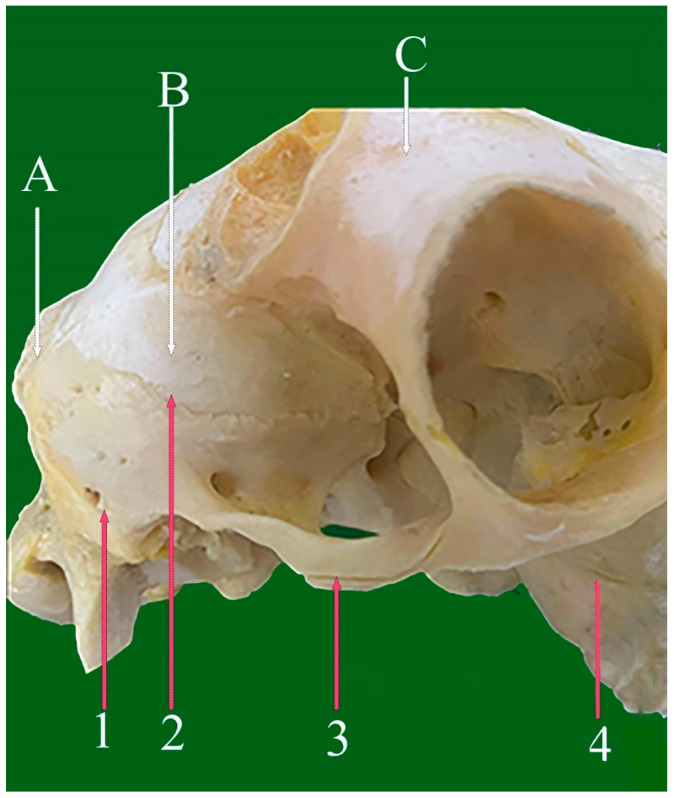
Lateral caudal view of sheep skull showing: 1—Occipito-parietal foliate suture. 2—Occipito-temporal foliata suture. 3—Zygomatico-temporal foliate suture. 4—Maxillary-zygomatic planar suture (horizontal part). A: Occipital bone. B: parietal bone. C: Frontal bone.

**Figure 7:**
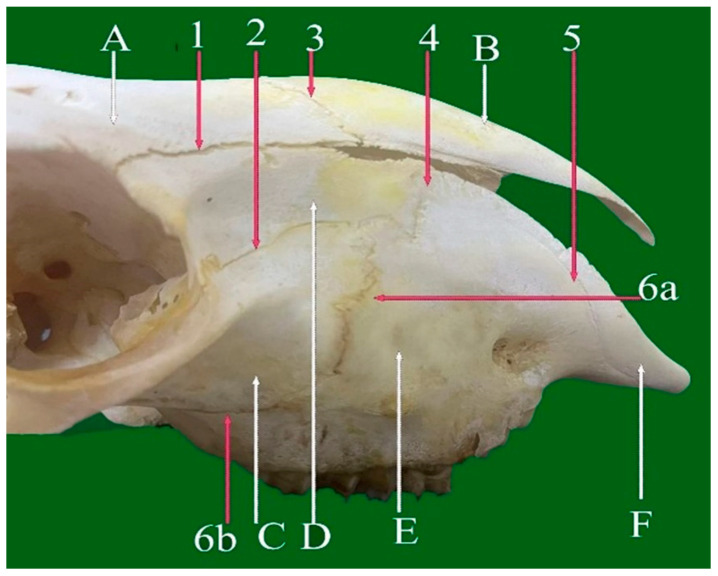
Lateral view of facial skull of sheep showing: 1—Fronto-lacrimal planar suture. 2—Lacrimo-maxillary serrata suture. 3—Frontonasal planar suture. 4—Lacrimo-maxillary serrate suture. 5—Incisive-maxillary (premaxilla) planar suture. 6a—Zygomatico-maxillary (vertical part) serrate suture. 6b—Zygomatico-maxillary (horizontal part) planar suture. A: Frontal bone. B: Nasal bone. C: Zygomatic bone. D: Lacrimal bone. E: Maxillary bone. F: Incisive bone.

#### 3.1.10. Median Palatal Planar Suture (Sutura Plana Palatina Mediana) (Figure 8(1))

Located along the midline of the hard palate, this suture results from the joint of the horizontal plates of the maxillary palatine processes. It extends rostro-caudally as a continuation of the intermaxillary fissure and terminates at the transverse palatal serrata suture.

**Figure 8:**
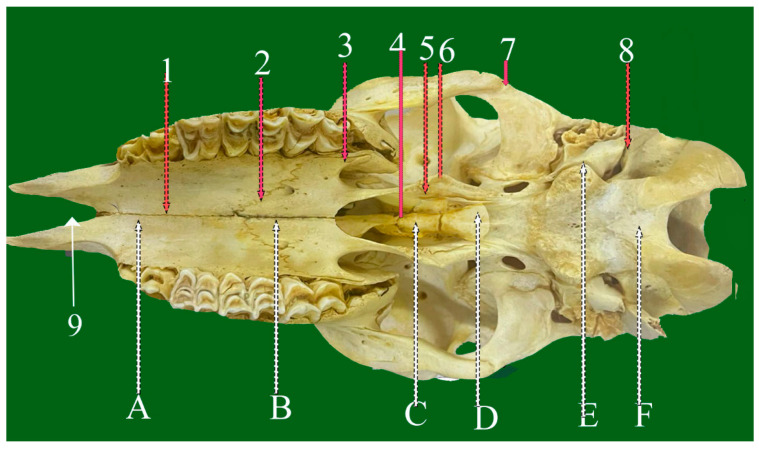
Ventral view of sheep skull showing: 1—Median palatine planar suture. 2—Transverse palatine serrate suture. 3—Palato-maxillary foliate suture. 4—Spheno-vomer foliate suture. 5—Pterygo-sphenoid planar suture. 6—Spheno-occipital planar suture. 7—Zygomatico-temporal foliate suture. 8—Temporal-mastoid foliate suture. 9—Intermaxillary fissure. A: Palatine process of maxilla bone. B: Palatine bone. C: Sphenoid bone. D: Pterygoid bone. E: Tympanic bull of petrous bone. F: Occipital bone.

#### 3.1.11. Pterygo-Sphenoid Planar Suture (Sutura Plana Pterygosphenoidalis) (Figure 8(5))

This suture connects the wings of the sphenoid bone with the pterygoid process of the palatine bone and also involves the maxillary bone. It follows a curved, vertically oriented course and plays a key role in the architecture of the pterygo-maxillary region.

#### 3.1.12. Spheno-Occipital Planar Suture (Sutura Plana Spheno-Occipitalis) (Figure 8(6) and Figure 9(2))

This suture is positioned at the cranial base, uniting the base–occipital and base–sphenoid bones. Its morphology contributes significantly to structural integrity at the cranio-cervical junction.

**Figure 9:**
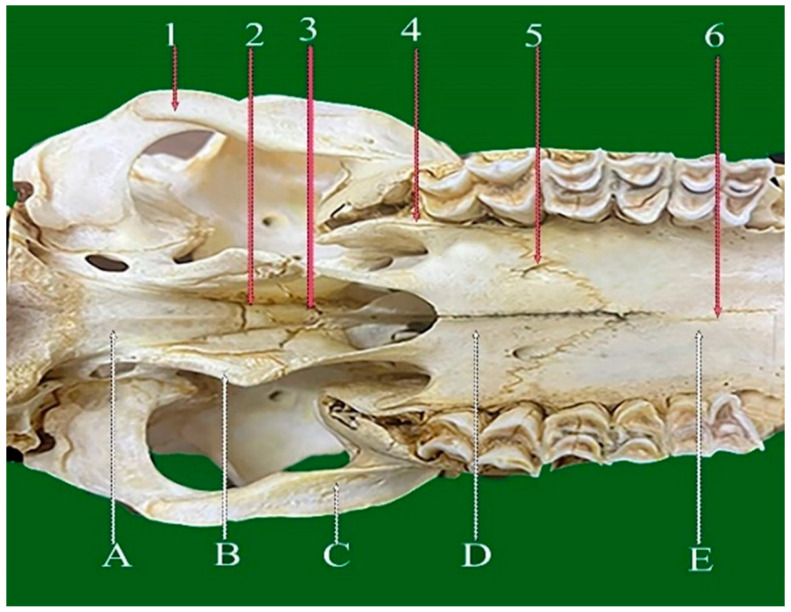
Ventral view of sheep skull showing: 1—Zygomatico-temporal foliate suture. 2—Spheno-occipital planar suture. 3—Spheno-vomer foliate suture. 4—Pterygo-palatine planar suture. 5—Transverse palatal serrate suture. 6—Median palatine planar suture. A: Occipital bone. B: Pterygoid bone. C: Temporal bone. D: Palatine bone. E: Maxillary bone.

### 3.2. Second Type: Cranial Serrate Sutures (Table 2)

#### 3.2.1. Frontal Serrate Suture (Sutura Serrata Frontalis) (Figure 1(4) and Figure 2(4))

This straight, elongated suture joins the two halves of the frontal bone in a sagittal orientation. The caudal two-thirds are distinctly serrated and originate from the coronal suture between the horn bases, while the rostral one-third transitions into a planar configuration and terminates at the internasal suture.

**Table 2:** List of Cranial Serrate Sutures with Corresponding Bone Articulations.

Second Type: Cranial Serrate Sutures			
No.	Serrate Suture Name	Bones Involved	Remarks
1	Frontal serrate suture	Right and left frontal bones	—
2	Fronto-parietal serrate (coronal) suture	Frontal bone and parietal bone	Also termed **coronal suture**
3	Lacrimo-maxillary serrate suture	Lacrimal bone and maxillary bone	—
4	Zygomatico-maxillary serrate suture (vertical part)	Zygomatic bone and maxillary bone	Vertical portion
5	Pterygo-palatine serrate suture	Pterygoid bone and palatine bone	—
6	Transverse palatal serrate suture	Palatine bone and maxillary bone	—

#### 3.2.2. Fronto-Parietal Serrate Suture (Sutura Serrata Fronto-Parietalis or Coronii) (Figure 1(5) and Figure 2(6))

It is termed as coronal; this slightly curved suture lies between the caudal border of the frontal bone and the rostral margins of the parietal bones. Extending laterally, it connects with the parieto-temporal suture within the zygomatic fossa and exhibits less pronounced serrations than the frontal suture.

#### 3.2.3. Lacrimo-Maxillary Serrate Suture (Sutura Serrata Lacrimo-Maxillaris) (Figure 3(6) and Figure 4(7))

This short horizontal suture joins the frontal process of the maxilla with the rostral margin of the lacrimal bone. It extends toward the nasolacrimo-maxillary fissure, where the nasal, lacrimal, and maxillary bones meet.

#### 3.2.4. Zygomatico-Maxillary Serrate Suture (Sutura Serrata Zygomatico-Maxillaris) (Vertical Part) (Figure 4(3) and Figure 7(6a))

Located approximately 2 cm rostral to the orbit, this vertical, short serrate suture connects part of the caudal border of the maxilla with the rostral edge of the zygomatic bone along the lateral aspect of the face. It extends dorsoventrally, enhancing the mechanical strength and stability of the orbital region.

#### 3.2.5. Pterygo-Palatine Serrate Suture (Pterygo-Palatina Serrate Suture) (Figure 5(1))

This is a robust, irregularly interdigitated joint between the vertical plate of the palatine bone and the pterygoid bone, functioning to firmly interlock and stabilize the adjoining bones.

#### 3.2.6. Transverse Palatal Serrate Suture (Sutura Serrata Palatina Transversa) (Figure 8(2), Figure 9(5) and Figure 10(3))

This suture unites the horizontal plates of the maxillary processes with the palatine bone in the caudal third of the hard palate, providing a strong and stable junction.

**Figure 10:**
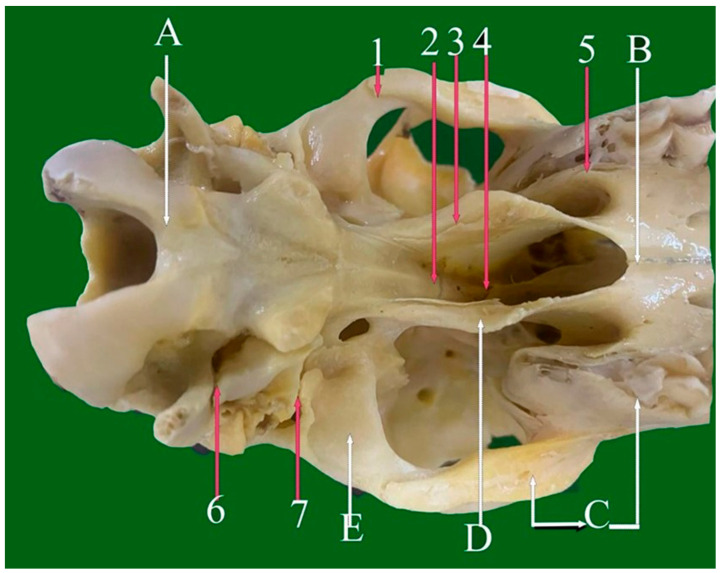
Ventrolateral view of sheep skull showing: 1—Zygomatico-temporal foliate suture. 2—Spheno-occipital planar suture. 3—Pterygo-palatine serrate suture. 4—Spheno-vomer foliate suture. 5—Palato-maxillary foliate suture. 6—Occipito-Mastoid (foliate Suture). 7—Parieto-mastoid suture. A: Occipital bone. B: Palatine bone. C: Maxillary bone. D: Temporal bone.E: Occipital bone.

### 3.3. Third Type: Cranial Squamosal Sutures (Table 3)

#### 3.3.1. Pterygo-Squamous Suture (Sutura Squamosal Pterygodius) (Figure 5(4))

This suture joins the petrous and squamous portions of the temporal bone, forming an interosseous connection between its medial and lateral components.

**Table 3:** List of Cranial Squamosal Sutures with Corresponding Bone Articulations.

Third Type: Cranial Squamosal Sutures			
No.	Squamosal Suture Name	Bones Involved	Remarks
1	Pterygo-squamous suture	Pterygoid bone and squamous part of the temporal bone	—
2	Parieto-temporal (squamosal) suture (*Sutura squamosa cranii*)	Parietal bone and squamous part of the temporal bone	

#### 3.3.2. Parieto-Temporal (Squamosal) Suture (Sutura Squamosal Cranii) (Figure 5(6) and Figure 6(2))

This curved suture connects the parietal and temporal bones on the lateral skull surface and continues caudally as the parieto-occipital suture. Together with the occipito-temporal suture, it contributes to the lateral aspect of the lambdoid suture.

### 3.4. Fourth Type: Cranial Foliate Sutures (Table 4)

#### 3.4.1. Naso-Frontal Foliate Suture (Sutura Foliate Naso-Frontalis) (Figure 1(2) and Figure 2(3))

The naso-frontal suture, which joins the nasal and frontal bones, is characteristically of the foliate type. It typically exhibits a V- or U-shaped configuration marked by overlapping, lamellar bony projections that create a distinctive leaf-like interdigitation. This sutural pattern is considered unique in sheep and is located on the dorsal aspect of the skull, conforming closely to the curvature of the cranial roof.

**Table 4:** List of Cranial Foliate Sutures with Cor-responding Bone Articulations.

Fourth Type: Cranial Foliate Sutures			
No.	Foliate Suture Name	Bones Involved	Remarks
1	Naso-frontal foliate suture	Nasal bone and frontal bone	—
2	Occipito-parietal foliate suture	Occipital bone and parietal bone	—
3	Zygomatico-temporal foliate suture	Zygomatic bone and temporal bone	—
4	Temporal-mastoid foliate suture	Squamous part and mastoid part of the temporal bone	Intra-temporal connection
5	Palato-maxillary foliate suture	Palatine bone and maxillary bone	—
6	Spheno-vomerine foliate suture	Sphenoid bone and vomer	—
7	Occipito-temporal (mastoid) foliate suture	Occipital bone and mastoid part of the temporal bone	—

#### 3.4.2. Occipito-Parietal Foliate Suture (Sutura Foliate Occipito-Parietalis) (Figure 2(7))

Located on the caudal skull surface, this transverse suture joins the caudal border of the parietal bone with the rostral margin of the occipital bone. It extends rostroventrally and transitions into the parieto-mastoid suture, forming part of the lambdoid complex.

#### 3.4.3. Zygomatico-Temporal Foliate Suture (Sutura Foliate Zygomatico-Temporalis) (Figure 10(5) and Figure 11(1))

This suture involves overlapping between the zygomatic process of the temporal bone and the temporal process of the zygomatic bone, contributing to the formation of the zygomatic arch.

**Figure 11:**
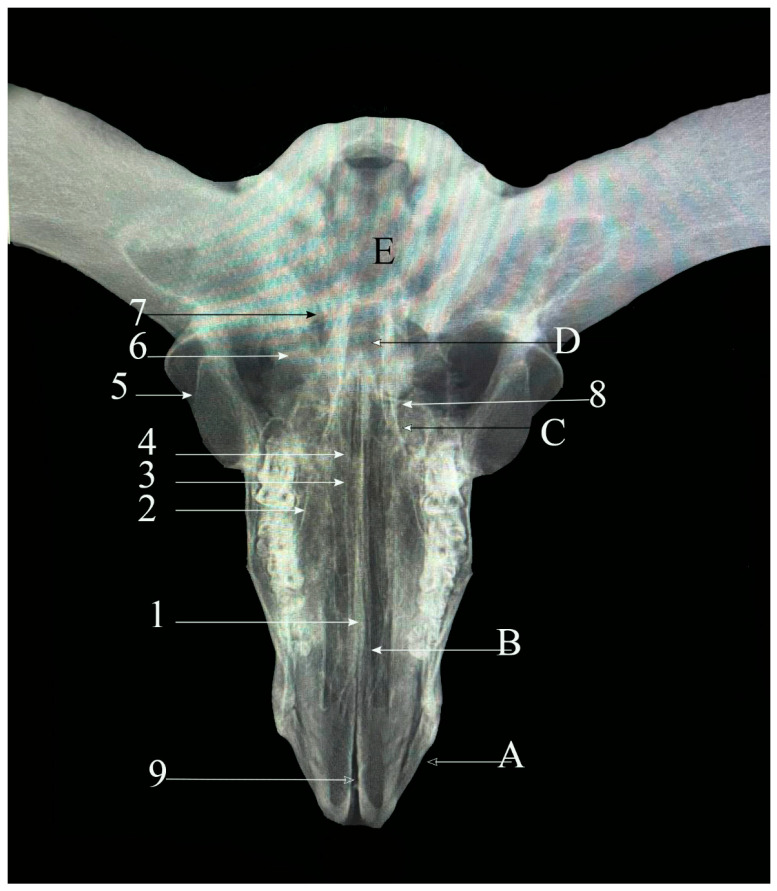
Ventrodorsal view of sheep skull showing: 1—Median palatine planar suture. 2—Palato-maxillary foliate suture. 3—Transverse palatine serrate suture. 4—Pterygosphenoid planar suture. 5—Zygomatico-temporal foliate suture. 6—Temporal-mastoid foliate suture. 7—Occipito-mastoid foliate suture. 8—Palato-maxillary serrate suture. 9—Intermaxillary fissure. A: Incisive bone: B: Palatine process of maxilla bone. C: Pterygoid bone. D: Sphenoid bone E: Occipital bone.

#### 3.4.4. Temporal-Mastoid Foliate Suture (Sutura Foliata Temporalis Mastoidea) (Figure 5(7), Figure 8(8) and Figure 11(6))

This obliquely oriented suture connects the occipital squama with the mastoid portion of the temporal bone and closely resembles the zygomaticotemporal suture in orientation.

#### 3.4.5. Palato-Maxillary Foliate Suture (Sutura Foliata Palate-Maxillaris) (Figure 8(3), Figure 11(2) and Figure 11(4))

Situated on the ventral skull surface, this suture unites the palatine bone and maxilla and extends caudally near the temporal fossa, enhancing regional stability.

#### 3.4.6. Spheno-Vomerine Foliate Suture (Sutura Foliate Spheno-Vomeriana) (Figure 8(4) and Figure 9(3))

This suture connects the presphenoid portion of the sphenoid bone with the alar processes of the vomer beneath the nasal septum. Its interlocking lamellae provide stability to both the cranial base and nasal cavity.

#### 3.4.7. Occipito-Temporal (Mastoid) Foliate Suture (Sutura Foliate Occipito-Temporalis) (Figure 6(4) and Figure 11(7))

Appearing as a groove or fissure at the caudo-lateral skull, this suture marks the articulation between the occipital bone and the mastoid process of the temporal bone.

This type of cranial suture in sheep is closely related to functional demands arising from nutrition and behavior. Nutritional requirements involving prolonged mastication favor plane sutures, which help stabilize the skull and dissipate compressive forces generated during chewing. In contrast, defensive and aggressive behaviors, such as head-butting, are associated with serrate sutures, which are structurally adapted for shock absorption and redistribution of impact forces, thereby reducing stress concentration and fracture risk. Squamous sutures, with their overlapping configuration, spread chewing and mild impact forces over a wider area, accommodate muscle forces, and protect deeper structures like the brain. Additionally, foliate sutures, acting together with increased skull thickness, contribute to overall cranial reinforcement and protection of the brain and its associated structures.

## 4. Discussion

Cranial sutures in mammalian skulls, including those of sheep, exhibit marked morphological diversity without differentiating between males and females. In the present study, males and females displayed comparable suture patterns, which may be attributed to similarities in the Najdi sheep breed and age among the examined specimens. Although frequently neglected in comparative analyses, cranial sutures demonstrate pronounced interspecific variation in their morphology. Suture length appeared to be proportional to overall skull dimensions, indicating a consistent pattern across samples and minimal intraspecific variation.

The observed diversity in suture morphology and configuration is closely associated with the structural characteristics of the adjoining cranial bones, where cranial sutures function as shock absorbers during impact [16]. This relationship has been previously emphasized by Standring [17], while Klein et al. [18] reported individual variability in suture positioning. Unlike squamosal or foliate sutures, where overlapping bones are common, planar sutures typically do not demonstrate thickening at the sites of articulation. However, De Pollack [19] noted that excessive growth along suture margins may occasionally result in premature fusion. In the present investigation, twenty-eight cranial sutures were identified in sheep, a finding consistent with reports in camels [20] and dogs [1], but contrasting with the higher numbers reported by Jones et al. [21] for mammalian skulls. Such discrepancies likely reflect interspecies differences in skull architecture and joint morphology. Similar observations have been reported in earlier anatomical studies involving sheep, goats, dogs, and camels [22,23].

In this study, sometimes, functional interpretation criteria of cranial sutures were based on clearly defined morphological features and corroborated by established biomechanical or comparative anatomical evidence. Specifically, interpretations were based on sutural complexity, degree of interdigitation, orientation relative to mechanical stress vectors, and documented associations with growth, load transmission, or cranial kinesis. Speculative or purely descriptive observations lacking functional relevance or empirical support were excluded to ensure that interpretations remained grounded in observable structure–function relationships.

Differences in suture morphology are strongly influenced by the shape and arrangement of adjacent cranial bones. In sheep, the internasal suture was observed to be planar and unfused, in agreement with Jashari et al. [5]. In contrast, fused internasal and nasal sutures have been described in other domestic species, where these bones are tightly integrated with surrounding structures [4,22,24]. Complete suture fusion has been reported in camels [20], whereas sheep demonstrate relatively looser articulations, underscoring clear species-specific differences in cranial suture patterns.

The naso-frontal suture in the present study displayed an intermediate configuration between a “V” and a “U” shape. This differs from the predominantly “U”-shaped pattern described in Mehraban sheep [25] and the “V”-shaped form reported in Bardhoka sheep [25]. In dogs, the naso-frontal suture has been described as larger and more serrated than in other species [1]. Comparable interspecies variation has also been documented in camels [15,20] and goats [26].

In sheep, it is notably longer than in other species. The fronto-lacrimal suture is typically well defined and relatively straight or slightly serrated, reflecting orbital stability [27]. In contrast, species such as the horse exhibit a more robust and functionally integrated fronto-lacrimal articulation due to the enlargement of the orbit and associated facial elongation [4]. The dog had a less prominent and more simplified suture [1]. These interspecies differences align with broader principles of cranial suture biology, while the lacrimal suture among domestic animals reflects functional adaptation [28], making it a valuable anatomical feature in comparative and applied veterinary research.

The naso-frontal foliate suture is positioned in sheep more rostrally than in other species, such as camel [20], dog [1], horse [4] and goat [26].

Notably, the present study demonstrated that the frontonasal suture exhibited greater interdigitation than other sutures, supporting the findings of Rafferty and Herring [26]. The frontonasal suture in sheep is similar to that of goats, characterized by a narrow, elongated nasal region and a moderately interdigitated suture [28]. In contrast, cattle exhibit a broader and more robust frontonasal suture due to larger cranial size and greater structural demands [29]. Compared with carnivores, sheep show a simpler and less interdigitated frontonasal suture [28]. Additionally, Allouch and Alshanbari [1] noted that the caudal extent of the naso-frontal suture in dogs is narrower than that of the maxilla-frontal suture.

Yahya et al. [30] reported a different pattern of incisive bone articulation in camels. In sheep, the premaxilla articulated rostro-ventrally between the maxillary and incisive bones without direct involvement of the nasal bone. This differs from the naso-maxillary-incisive notch described in dogs by Buzek et al. [31], a feature absent in sheep, further emphasizing interspecies variation.

We agree with Yi et al. [32], who reported distinct anatomical features on the fissura nasolacrimalis and fissura nasomaxillaris in the skull of the Korean native goat, which were classified into four types based on variations in the articulations of the surrounding bones according to their study.

Overall, the present findings are consistent with Standing [17] in demonstrating that cranial bones are interconnected by sutures that vary in form and complexity. In contrast to Casanova [33], who reported asymmetry in several sutures, including the occipito-temporal region, all sutures examined in this study were bilaterally symmetrical. A comprehensive understanding of cranial sutures is essential for the accurate diagnosis of congenital anomalies and for distinguishing normal anatomical features from skull fractures [34]. Furthermore, their critical role in craniofacial growth and development has been widely recognized [35].

Comparisons between sheep and camels reveal clear evolutionary differences in the timing and rate of suture closure. Sheep tend to exhibit a relative acceleration in certain suture zones associated with horn support and behavioral competition [36], while camels maintain a higher degree of cranial flexibility for a longer period [20], which is interpreted as an adaptation to different environmental and functional stresses.

Balcarcel et al. [37] reported significant differences in mean cranial shape between wild and domestic goats, as well as among specific breeds. Their findings indicate that domestication disrupts the ancestral cranial form, showing that even low-intensity selective pressures can produce notable morphological changes. In light of this, the Najdi breed of sheep was selected for the present study so that its cranial characteristics can later be compared with those of other sheep breeds.

## 5. Conclusions

This study provides a comprehensive examination of ovine cranial sutures, including serrate, planar, squamosal, and foliate types, highlighting morphological characteristics that differentiate them from those of other mammalian species. By documenting the precise form, orientation, and interdigitation of these sutures, the research enhances our understanding of species-specific cranial architecture. Cranial sutures serve not only as structural connectors between skull bones but also as critical anatomical landmarks, reflecting patterns of growth and development within the neuro-cranium. Their detailed mapping is particularly valuable in clinical and surgical contexts, including neurosurgical procedures, where the accurate identification of sutures informs the safe navigation of vital structures such as the brain, major arteries, and venous sinuses. Moreover, insights gained from ovine sutures contribute to comparative anatomy, offering a framework for understanding evolutionary adaptations, interspecies variation, and functional biomechanics of the skull. Collectively, these findings underscore the importance of cranial sutures as both structural and clinical reference points in veterinary and biomedical research.

This study has several limitations. The sample size and potential lack of diversity in breed, age, and sex may restrict the generalizability of the findings. In addition, the study relies primarily on qualitative morphological descriptions without detailed morphometric or statistical analyses, which may affect objectivity and reproducibility.

## Data Availability

The original contributions presented in this study are included in the article. Further inquiries can be directed to the corresponding author.
